# The amount of penicillin needed to prevent mother-to-child transmission of syphilis

**DOI:** 10.2471/BLT.16.173310

**Published:** 2016-08-01

**Authors:** Melanie M Taylor, Xiulei Zhang, Stephen Nurse-Findlay, Lisa Hedman, James Kiarie

**Affiliations:** a Department of Reproductive Health, World Health Organization, avenue Appia 20, 1211 Geneva 27, Switzerland.; b Centre for Tuberculosis Control, Shandong Provincial Chest Hospital, Jinan, China.; c Department of Essential Medicines and Health Products, World Health Organization, Geneva, Switzerland.

Syphilis is unique among sexually transmitted diseases in that it remains curable (with minimal reports of resistance) with a single dose of penicillin, formulated for this purpose as long-acting benzathine penicillin.^1^^,^^2^ Pregnant women with untreated syphilis experience adverse birth outcomes in over half of untreated pregnancies. These outcomes include stillbirth, organ deformities, prematurity and neonatal death.^3^ The World Health Organization (WHO) estimates that 930 000 pregnant women have probable active syphilis (transmissible during pregnancy) annually which results in approximately 350 000 adverse birth outcomes per year.^4^ Most maternal syphilis cases and adverse pregnancy outcomes occur in low- and middle-income countries, and more than half occur in sub-Saharan African countries.^4^ WHO estimates that global syphilis prevalence is 0.5% (95% uncertainty interval, UI: 0.4–0.6%) which corresponds to a global incidence of 5.6 million (95% UI: 4–8 million) syphilis cases per year among people aged 15–49 years.^5^ From these estimates, it is possible to calculate the amount of penicillin needed for syphilis treatment on a global scale. However, the difference between the global needs and what is currently produced is not possible to quantify as there is no global monitoring of availability.

In May 2016, benzathine penicillin was recognized by the 69th World Health Assembly as an essential medicine that has been in short supply for several years. These shortages have affected treatment and prevention of congenital syphilis.^6^ A lack of benzathine penicillin can result in pregnant women with syphilis receiving ineffective or no treatment.^7^^–^^10^ Benzathine penicillin is a generic injectable with very few global manufacturers and is therefore at high risk for inventory stock out and shortages at the point-of-care. WHO has received reports of stock outs of benzathine penicillin from antenatal care representatives and providers in high morbidity countries from three WHO regions.^11^ Shortages during 2015 and 2016 at the manufacture and supply levels have been reported in the region of the Americas.^12^^–^^14^

An estimated 5.6 million doses of 2.4 million units of benzathine penicillin are needed annually to treat all syphilis cases.^5^ Of these, 930 000 doses are needed to treat pregnant women with syphilis early in pregnancy to prevent all cases of congenital syphilis.^4^ These separate estimates indicate an acute need for expanded access to benzathine penicillin in countries with high rates of adult syphilis and ongoing improvements in syphilis screening during antenatal care. WHO advocates use of these estimates in discussions at the manufacture and distribution levels to ensure a reliable supply of benzathine penicillin in all countries with cases of adult syphilis.

Global demand for this formulation of penicillin has not been consistently and adequately quantified. Many people with syphilis do not develop pathognomic signs or symptoms and do not receive a diagnosis because they are unaware of infection or are unable to access health care and/or diagnostic tests. Therefore, these estimates of penicillin requirements are greater than the number of adults who will have been diagnosed with syphilis. These estimates do not include the treatment of congenital syphilis in infants as such regimens require different doses of aqueous, procaine, or benzathine penicillin formulations.^1^^,^^2^

As a result of increasing need and reported shortages, and in response to the directives from the 69th World Health Assembly, WHO, in partnership with other stakeholders, is doing a market analysis that will include evaluation of production, demand, supply and procurement practices at manufacture, country and regional levels. Results of this analysis will be used to further refine estimates of benzathine penicillin demand, improve availability and develop interventions to improve supply management and procurement.

WHO’s plan for the elimination of mother-to-child transmission of HIV and syphilis includes targets of 95% of pregnant women receiving antenatal care and receiving syphilis testing during antenatal care, and 95% of women diagnosed with syphilis during pregnancy being treated.^15^ Elimination targets for syphilis are achievable given that benzathine penicillin, the only recommended treatment for syphilis occurring in pregnant women, can cure maternal syphilis and prevent adverse birth outcomes related to congenital syphilis if provided early in pregnancy.^1^^,^^2^ Countries seeking to expand and improve prenatal care, reduce adverse pregnancy outcomes, and achieve congenital syphilis elimination targets must have access to a secure supply of benzathine penicillin. Equally, manufacturers need a reasonable global needs estimate as part of the business case required to justify any changes to their manufacturing processes. These WHO estimates of penicillin needs can be used along with estimates of demand to adapt manufacturing practices to expand access to benzathine penicillin.
